# Reflections from Chinese and Japanese Physicians on Medical Disputes

**DOI:** 10.1007/s41649-024-00294-5

**Published:** 2024-08-13

**Authors:** Hua Xu, Yining Ruan, Taketoshi Okita, Masao Tabata, Yasuhiro Kadooka, Atsushi Asai

**Affiliations:** 1 Department of Medical Ethics, Tohoku University Graduate School of Medicine, 2-1, Seiryocho, Aoba-Ku, Sendai, Miyagi Prefecture 980-8575 Japan; 2 Division of Philosophy and Ethics, Department of Culture and Medicine, School of Medicine, Shiga University of Medical Science, Otsu, Japan; 3 Patient Safety Management Office, Tohoku University Hospital, Sendai, Japan; 4 Department of Bioethics, Faculty of Life Sciences, Kumamoto University Graduate School of Medicine, Kumamoto, Japan

**Keywords:** Cross-cultural study, Case analysis, Medical dispute, Professionalism, China, Japan

## Abstract

Physician–patient disputes are a major problem in healthcare. Physician–patient conflicts, workplace violence, and direct involvement in disputes have a significant negative impact on the well-being of physicians. China and Japan have similar cultures but differing healthcare systems. The present study aimed to examine and compare the experiences and perceptions of Chinese and Japanese physicians regarding medical disputes. Qualitative descriptive content analysis was performed for 18 cases from each country to assess the major issues involved in each case and their impact on the physicians. Common issues in medical disputes for both countries included monetary motives of patients and/or families, violence/threats from patients and/or families, the inability of patients and/or families to understand the risk of complications, and the uncertainties of medicine. The serious impact of medical disputes on the mental health and professionalism of physicians was also an issue shared by physicians of both countries. There were, however, differences in the magnitude and frequency of these issues between the two countries. Pre-existing distrust of physicians among patients and/or families was noted only by Chinese physicians, and insufficient information disclosure by physicians was noted only by Japanese physicians. In conclusion, there were similarities and differences between the two countries in the perceptions of physicians regarding medical disputes. Our analysis revealed differing healthcare situations due to cultural and institutional differences as well as universal problems intrinsic to medicine. Based on our results, we propose several key principles to improve the physician–patient relationship.

## Introduction

Physician–patient conflict is a major issue in modern healthcare. Several studies have found that the number of medical disputes and violent incidents in medical institutions caused by conflicts, dissatisfaction in the physician–patient relationship, and medical accidents has been on the rise in many countries including China and Japan (Citizens’ Association for the Disclosure and Publication of Medical Information 2021; Iwao et al. 2013; Liu et al. 2022; Loue 2020; Montgomery et al. 2020; Saitama Newspaper 2022; Yoshimura et al. 2022). From 2009 to 2018, 295 severe medical violence events were reported on social media in China, in which 362 doctors were injured and 24 lost their lives (Wang and Du 2023). Confrontation and hostility between physicians and patients in the healthcare setting, and responses to medical incidents, particularly adverse events involving medical errors, have a significant negative impact on the well-being of medical professionals, including excessive workload, exhaustion, stress, demoralization, depression, and sleep deprivation (Citizens’ Association for the Disclosure and Publication of Medical Information 2021; Iwao et al. 2013; Liu et al. 2022; Loue 2020; Yin et al. 2023; Hanganu et al. 2020; Montgomery et al. 2020; Saitama Newspaper 2022; Sahebi et al. 2022; Varghese et al. 2021; Wang et al. 2022; Yoshimura et al. 2022, Zhu et al. 2022).

Various causes underlie physician–patient conflicts. For instance, on the physician side, these include inappropriate attitudes of physicians such as arrogance and critical behavior toward patients, inadequate information provision by physicians, inadequate decision-making processes, poor clinical skills and care, occurrence of side effects and complications, medical accidents, medical errors and their concealment, dishonesty, and lack of a sense of responsibility (Citizens’ Association for the Disclosure and Publication of Medical Information 2021; Editorial 2019; Wachter and Gupta 2018; Wang et al. 2022). Underlying factors on the patient side include, for example, a misunderstanding of medical information, dissatisfaction arising from excessively high expectations of treatment outcomes, lack of medical knowledge, confusion between complications and medical errors, and distrust and suspicion of physicians and medical institutions as well as of the medical system in general. Other factors include the influence of mass media coverage, waiting times, and high medical costs (Citizens’ Association for the Disclosure and Publication of Medical Information 2021; Hanganu et al. 2020). The cover-up of medical errors due to fear of litigation also is a major ethical and socio-legal problem, and in this context, a culture of honesty is important (Ceriani-Cernadas 2017; Sabroe et al. 2021; Wachter and Gupta 2018).

China and Japan are in the same East Asian region and have similar cultures and relationships which are influenced by Confucianism and Buddhism, although their medical systems and health insurance schemes differ as shown in Table 1 (EPILOGI Editorial Office 2015; Fen 2021; GBD 2016 Healthcare Access and Quality Collaborators 2016; Katayama 2019; Ma 2022; Ministry of Economy, Trade and Industry 2022; Fen 2021, Sato et al. 2022; National Health Commission of the People’s Republic of China 2022; Wei 2016; Xu and Oshima 2020; Xinda and Xintong 2023). Yet, aside from their common Asian ethnicity, Chinese character culture, rice as the staple food, and influence of Confucianism, they reportedly have little else in common (Kondo 2022). To our knowledge, no qualitative study has directly compared cases of medical disputes in China and Japan and their impact on physicians.

**Table 1:** Summary of the healthcare background of China and Japan

Category	China	Japan
Per capital health care costs (2018 US$)	501	4267
Number of hospital beds per 1000 people	6.46 (2020)	12.1 (2019)
Number of physicians (except dentists) per 10,000 people	25 (2020)	24.8 (2018)
Number of nurses per 10,000 people	33.4 (2020)	127 (2018)
Universal health insurance system	Yes	Yes
Medical insurance cover rate	95%	99%
Co-payment rate	It depends (5–50%)	30% in general 20% (70–74 yo and the low-income), 10% (≧75 y)
High-cost medical expenses benefit	It depends (up to 85–95%) (many regions have benefit limits)	Yes
Benefit limit	Yes (not in some regions)	No
Percentage of medical expenses to be paid out-of-pocket (percentage of GDP)	30.9%	12.8%
Free access to medical facility	Yes	Yes
Ambulance fees (including helicopter fee)	Yes	No
Differences in healthcare insurance system by region (rate of medical expense benefit)	Yes	No
Cross-regional insurance coverage	Yes	Yes
Differences in initial consultation fees by physician rank	Yes	No
Differences in reimbursement rate by hospital rank※	Yes	No
Healthcare Access and Quality (HAQ) Index rank among 195 countries	48th	12th
Annual incomes of physician in hospitals (JP yen)	About 1,280,000	About 12,000,000
Annual incomes of general public (JP yen)	About 550,000	About 4,140,000

※Hospital type (rank): primary healthcare institution (A in China), secondary hospital (B in China), and university hospital (C in China)

Comparative research of China and Japan is informative for a number of reasons. First, according to recent reports as mentioned above (Iwao et al. 2013; Wang and Du 2023), the state of medical disputes is more dire in China than in Japan. As there are similarities and differences between the two countries in terms of the healthcare system and environment, a comparative study can identify factors that are strongly related to the occurrence of medical disputes. Second, such studies can demonstrate how differences in cultural and social aspects, interpersonal differences, and historical changes in the two countries are related to medical disputes. Third, experiences and perceptions of physicians from both countries can provide useful information for people of both countries to learn about and from each other. For instance, results from such studies may provide insight into means for conflict resolution in each country. Finally, it is also expected that there will be more opportunities to access each other’s medical systems from citizen-level exchanges between China and Japan. Therefore, we believe that it is necessary to deepen our understanding by comparing the current state of medical care in both countries.

Accordingly, the present study aimed to clarify and compare perceptions of Chinese and Japanese physicians regarding their experiences generally, and regarding medical disputes, more particularly. Through a cross-cultural comparative qualitative case series analysis, we explored obstacles and facilitators for realizing mutually trustful, respectful, and harmonious physician–patient relationships and sought to better understand medical disputes in both countries from an ethical perspective. We also discuss the impact of medical disputes on medical professionalism by providing narratives on everyday experiences from physicians.

We note that the focus of this study is not on the cases themselves as experienced by physicians, but rather the actions of those involved at critical moments, especially patients and/or their families, and the physicians’ reflections on such experiences. Although we cannot provide any definitive conclusions given the preliminary, exploratory, and qualitative nature of the study, our findings raise several important issues that require urgent attention in order to improve the current state of medical disputes and achieve more harmonious physician–patient relationships. To this end, we propose several key principles to improve the physician–patient relationship.

## Methods

The present study is part of a China-Japan comparative study on physician–patient relations, physician–patient disputes, and physician well-being. In the main study, semi-structured interviews were conducted with 20 Chinese physicians and 20 Japanese physicians regarding physician–patient relationships, decision-making models, current state of medical disputes, and physician well-being in both countries, using the same questions. Physicians in both countries were sampled through the researchers’ personal networks. Snowball sampling was also used for some participants. All participating physicians were active working physicians who had already completed their clinical training by the time of the interviews. Interviews were conducted in both countries over a 2-month period spanning March and April 2023. This study was approved by the Ethics Committee of Tohoku University Graduate School of Medicine on January 26, 2023 (2022–1-886). At the time of conducting the interviews, the method and intent of the study were explained, and all participants provided written informed consent. All participants were informed that the contents of the interviews would be recorded and that their statements would be reported anonymously. No honorarium was paid for participating in the study. The medical dispute cases presented here are those mentioned during the interviews. These examples are provided based on physicians’ responses to the question, “Describe your experiences regarding physician–patient conflicts (all conflicts between patients and medical professionals, including confrontations, disputes, aggressive attitudes, violence, lawsuits, etc.).” Many of the physicians also provided their reflections on these medical dispute experiences.

Qualitative descriptive content analysis of the case series was used to identify major issues and themes involved in the cases and their impact on the physicians in a qualitative and inductive manner (Sandelowski 2000; Satu and Kyngas 2008; Gibbs 2018). According to Sandelowski, qualitative description is a complete and valued end-product in itself rather than an entry point into other qualitative studies such as grounded theory, phenomenology, or ethnography, and qualitative descriptive studies have a long history in many academic fields (Sandelowski 2000). Qualitative descriptive studies can offer a comprehensive summary of an event in everyday terms of those events and are the method of choice when straight descriptions of phenomena are desired and the study method is amenable to obtaining straight and largely unadorned answers to questions of special relevance to practitioners and policy makers, including questions relating to people’s concerns, responses, reasons, and influencing factors on a given issue (Sandelowski 2000). Qualitative description is less interpretative than the above-mentioned methods and does not require a conceptual or other otherwise highly abstract rendering of data (Sandelowski 2000). Researchers conducting qualitative descriptive studies stay closer to their data; moreover, qualitative descriptive studies may be the least theoretical of the spectrum of qualitative approaches and are the least encumbered by pre-existing theoretical and philosophical commitments (Sandelowski 2000). However, qualitative descriptive studies are not atheoretical and they tend to follow the philosophical foundations of natural inquiry, usually describing a participant’s experience directly or presenting an event in simple language as free of artifice as possible in the artifice-laden enterprise known as conducting research (Ma et al. 2023; Sandelowski 2000, 2010).

The initial steps of all analysis were conducted by Hua Xu (H.X.) and Atsushi Asai (A.A.), who then shared their results with all researchers/authors and discussed them until a consensus was reached on the contents. More specifically, the following steps were taken. First, from the verbatim transcripts of each interview, relevant cases regarding physicians’ experiences and statements on medical dispute cases were extracted. A total of 36 cases were extracted, with 18 each from Chinese and Japanese physicians. Second, the researchers reviewed the case-related sections several times and extracted the following descriptions from the physicians’ statements: (1) what happened to the patient in the case, (2) reactions of the patient and/or patient’s family after the event, (3) the physician’s reflection on the reactions of the patient and/or patient’s family, and (4) the impact of the medical dispute on the physician. Third, the descriptions were replaced with shorter and more abstract expressions. The same process was continued iteratively and the researchers confirmed and agreed with the appropriateness of the summary descriptions. Descriptions (1) and (2) are summarized in Table 3. Finally, similar summary descriptions were grouped together, and brief themes were assigned to them to commonly represent all descriptions in the same groups (Table 4). All researchers reached a consensus on all outcomes of the above qualitative, inductive, and descriptive content analysis for the case series.

## Results

Table 2 summarizes background information of the participating physicians. Table 3 briefly summarizes the experiences described by the physicians, together with the reactions of the patients and/or their families. Some physicians did not provide specific examples of their experiences with medical disputes, while others provided several experiences. We focused on cases which had a complete overall picture through clear descriptions of the physicians’ experiences. It should be noted that the motives and feelings of the patients and their families (or the bereaved family if the patient died), as well as their social circumstances, were unknown. In this regard, Table 3 describes events and actions taken by patients and/or their families based solely on statements made by the physicians during the interviews.

**Table 2:** Demographic characteristic of the study participants

Category	China	Japan
	No. (%) or mean ± SD (*N* = 20)	No. (%) or mean ± SD (*N* = 20)
Interview time mean (min)	50 (27–148)	52 (37–80)
Gender		
Female	4 (20)	3 (15)
Male	16 (80)	17 (85)
Age		
20 s	1 (5)	0
30 s	4 (20)	1 (5)
40 s	12 (60)	5 (25)
≧50 s	3 (15)	14 (70)
Working experience	16 ± 8	29 ± 13
5–9 y	4 (20)	0
10–19 y	12 (60)	2 (10)
20–29 y	2 (10)	9 (45)
≧30 y	2 (10)	9 (45)
Hospital type		
Primary healthcare institution		5 (25)
Secondary hospital		6 (30)
University hospital	20 (100)	9 (45)
Specialty		
Internal medicine	2 (10)	14 (70)
ER	3 (15)	
Surgery	14 (70)	6 (30)
ICU	1 (5)	
Conflict experience with patient and/or its family		
Aggressive attitudes/words	19 (95)	2 (10)
Physical attack	6 (30)	1 (5)
Personal/professional involvement in formal dispute process	17 (85)	7 (35)

**Table 3:** Brief case description and reactions of patients and/or their families

Case brief (participant ID)
A pregnant patient died suddenly. Afterwards, many family members stormed the physician’s office. (C2)
A physician made a proper medical judgment and decided that surgical treatment for the patient was not necessary. The patient did not trust the decision, attacked the physician, and spread rumors about the physician. (C3)
A physician made a medically appropriate decision and recommended the patient to visit another department. The patient mistook the decision as a refusal of medical treatment and reported it to the Mayer Hotline. (C4)
A critically ill patient died in the hospital. The patient’s family accused the physician and grabbed her. They followed the physician for some time afterwards. (C6)
A physician had performed certain tests on the inpatient as medically appropriate. The patient’s family claimed that the tests were unnecessary and requested a refund of the cost of the tests. (C7)
A drunk trauma patient took it out on his physician. He was angry that the expensive test ordered by another physician to diagnose his abdominal pain, which he had received a month earlier, had been wasted. (C8)
An adverse event occurred in the newborn immediately after delivery. Although the physician only admitted the mother of the child to the hospital and she was not involved in the birth of that child at all, the patient’s family kept following the physician. (C9)
A patient’s family discharged a seriously ill patient against medical advice and the patient died at home. The family sued the physician, knowing that there was no medical error. (C10)
A patient died after being admitted urgently to a hospital when becoming seriously ill after hesitating to see a physician for a long time. The family indicated that the physician falsified the cause of death in order to increase the insurance benefit, and at the same time sued the physician for medical malpractice. (C11)
A young physician objected to the patient’s treatment plan. The patient’s family did not trust her opinion because of the physician’s age and accused the physician of delaying treatment. (C13)
A young physician pointed out the real disease that had been missed. The patient’s family did not trust the opinion of the physician because she was young and tried to refuse the necessary treatment. (C13)
A young physician was sued by family members in a case in which he was not directly involved in the treatment of the patient. (C14)
A family sued the physician, despite knowing that there was no medical error. (C15)
A postoperative patient developed a subcutaneous tissue infection due to self-extraction of a drainage tube. The family refused to acknowledge the self-extraction, followed the physician for several days, and filed a lawsuit. (C16)
The scene was chaos as patients with minor illnesses who did not need emergency care flooded the emergency department. (C17)
Regulations at the facility prevented the use of certain antibiotics and the patient died. The family withdrew from the lawsuit after the physician explained the situation and family members were persuaded. (C18)
A patient who did not return to the hospital died at home, contrary to the physician’s instructions to see him immediately if symptoms persisted. The bereaved family followed the physician around for several days and repeatedly harassed the physician in various ways. (C19)
A patient and the patient’s family were not satisfied with the effectiveness of inpatient treatment, despite careful prior explanations. The patient and family threatened to sue the physician. (C20)
A patient undergoing invasive treatment died from complications. One of the bereaved family members who had not been fully informed about the complications attempted to hit the physician. (J1)
Failure to diagnose a fatal disease resulted in the death of a young patient. The physician apologized and the family accepted the apology. (J2)
A patient unilaterally refused to continue the medical relationship with the physician. (J3)
A patient suffered cardiopulmonary arrest due to complications from an examination, and an on-call physician performed cardiopulmonary resuscitation. (J4)
An angry patient came to the hospital (details unknown). The physician was worried that she might be sued. (J5)
A patient died as a result of complications from the test. The physicians who carried out the test were not available and a physician explained the circumstances to the family as a patient safety officer. (J7)
A physician missed an abnormality noted in a laboratory report and the disease was left untreated for a year. A representative of the hospital visited the patient’s home and apologized on behalf of the hospital. The patient accepted the apology. Priority treatment and compensation was offered. (J8)
A patient died as a result of complications from an invasive examination. The family tried to sue the patient’s physician, not the physician in charge of the test, but the case did not actually go to court. (J9)
A patient and the patient’s family were not satisfied with the occurrence of postoperative complications and blamed the physician, despite having agreed to the operation after receiving a detailed explanation beforehand. (J11)
A foreign patient did not communicate well enough in Japanese with the physician, and the patient became furious with the physician, partly due to anxiety about the potential illness. (J12)
A patient and the patient’s family were not convinced of the outcomes of lengthy inpatient treatment because an attending physician failed to provide adequate explanations in advance. (J13)
A patient’s family left the hospital seemingly satisfied with the physician’s explanation of the circumstances of the patient’s death. Sometime later, they sued the physician for medical malpractice. (J14)
Symptoms worsened after painful treatment. A patient believed that the painful treatment had worsened his symptoms and sued the physician. (J15)
A postoperative cancer patient had complications and died after hospitalization. The bereaved family was not satisfied with the physician’s explanation that there had been no problems with the course of treatment and was furious, urging the physician to “show some sincerity.” (J16)
Cancer recurred in a postoperative cancer patient. The patient believed that the recurrence was due to a late surgical date set by the physician without consulting him carefully. (J17)
A patient mistook the name of a disease listed as one of the differential diagnoses for a definite diagnosis and believed that the physician had misdiagnosed him. Unsatisfied with the subsequent explanation, the patient sued the hospital. (J18)
After an intravenous infusion a patient complained of symptoms that could not be explained by neuropathy. The physician foresaw potential trouble with him and referred him to a specialist at another hospital to ensure there were no abnormalities. (J19)
The surgeon downplayed the complications that occurred after the operation and did not adequately explain the situation to the patient/family. The patient subsequently went into shock and the family sued the physician. (J20)

Chinese physicians are represented by “C + No.”; Japanese physicians are represented by “J + No.”

Table 4 shows cross-cultural similarities and differences in major issues as well as themes pertaining to medical disputes in China and Japan extracted from 36 cases, with categories listed in the order of the number of mentions by Chinese physicians. Many of the issues raised by the physicians could be considered potential causes which led to a serious conflict. Below, the behavior of patients and/or their families in medical disputes (in the order of the themes presented in Table 4), the reflections of physicians on these behaviors, and the impact of medical dispute experiences on the physicians are discussed. Where appropriate, relevant and representative statements made by physicians are quoted.

**Table 4:** Cross-cultural similarities and differences in major issues involved in medical disputes between China and Japan

Issues mentioned by participants	China	Japan
Monetary motives of patients and/or their families	10*	2*
Violence/threats by patients and/or their families	9	5
Strong distrust existing from before seeing a physician in patients and/or their families	6	0
Inability of patients to understand the risk of complications and the uncertainties of medicine (differences in perceptions)	2	3
Concentration of patients in large hospitals	1	0
Inadequate explanation by physicians of treatment plans or potential complications to patients and/or their families	0	4
Misinterpretation of physician statements by patients	0	2
Unilateral discontinuation of the physician–patient relationship by patients	0	1
Difficulty understanding the patient’s state of mind by physicians (delayed lawsuits)	0	1

^*^Number of cases in which issues were mentioned by participants

### Monetary Motives of Patients and/or Their Families

In both countries, physicians described the explicit or implicit monetary motives of patients and/or their families. For instance, Chinese physicians noted that patients and/or their families attacked physicians and spread bad rumors about them when they were dissatisfied with the medical bills. Even in cases where the bereaved family admitted that the healthcare provider was not at fault, money was the sole reason for the dispute. Bereaved families did not admit fault on the part of patients and initiated disputes for financial gain. They also sued physicians who were not directly involved in the medical accidents which led to the disputes. One bereaved family pressured physicians to change their statements about the cause of death in order to increase payments from the insurance company. Family members of one patient who had not shown up at medical appointments showed up only when demanding money. Some Chinese physicians noted that this behavior of family members can be attributed entirely to poverty.

“*No reasonable grounds were presented in the complaint materials. The family members expressed very clearly that they just wanted money*.” (Chinese physician 10).

Japanese physicians stated that one patient’s bereaved family was furious with the hospital’s explanation that there was nothing wrong with the course of treatment, and urged the hospital to “show some sincerity.” In this case, “show some sincerity” was suspected to mean a payment. Another suspicious emergency patient complained that an intravenous drip into his forearm had somehow caused him to lose the ability to lift his shoulder. In that case, there was no overt monetary motive, but rather only a suspicion.

“*The bereaved family recognized that the patient had died due to inappropriate treatment for hypokalemia and the delay in treatment and surgery for the bowel obstruction. Outraged when the hospital explained that there was no problem with the course of treatment, the bereaved family urged us to ‘show some sincerity*.’” (Japanese physician 16).

### Violence/Threats by Patients and/or Their Families

In both countries, physicians mentioned various types of violence and/or threats from patients and/or their families. Chinese physicians stated that violence and threats by family members occurred when the family members believed that the patients died due to medical errors, when they were dissatisfied with the course and/or outcomes of treatments, or when they wanted to make their case for compensation. Their actions included intrusion into the physician’s office by multiple people, stalking/following, threatening behaviors, retaliatory actions (spreading bad publicity), physical violence, and verbal intimidation. Some patients also physically attacked the physicians.

“*If his (the drunk patient’s) leader hadn’t appeared at that time, I would have been beaten, and the emergency department space was too small to make an escape*.” (Chinese physician 8).

“*After the patient died, the family member quarreled with me, pulled me into the department, and followed me later*.” (Chinese physician 6).

As for Japanese physicians, there was one case in which a family member of a patient who died suddenly almost attacked the physician. There was also a case in which a family member persistently abused the physician because the patient did not improve as the family had hoped. In another case, a patient became mentally anxious due to strong fears that he might be afflicted with a serious disease, and took out his anger on the physician with whom he could not communicate well. None of the Japanese physicians, however, described any cases of actual violence.

“*I begged the son to stop the violence and listen to me, and carefully explained the situation in detail*.” (Japanese physician 1).

### Strong Distrust Existing from Before Seeing a Physician in Patients and/or Their Families

Many Chinese physicians stated that patients and/or their families did not believe the diagnosis at all from the beginning. In other words, there was distrust of physicians and/or medicine as a basic premise or preexisting distrust among those who need healthcare. In one example, a physician’s judgment was not trusted because of her young age. Specifically, the patient’s family did not believe the physician’s explanation regarding the severity of the patient’s condition and did not believe that the tests recommended and performed by the physician were necessary. There was a perceived distrust of tests being performed for the physician’s financial benefit. Another patient made an unnecessary visit to the emergency room and felt denied treatment when an ER physician said that it was nothing serious, and the patient complained to the authorities the next day. It was also noted that people did not trust physicians they did not know and many Chinese people want to be seen by physicians who they know personally. There were no descriptions from Japanese physicians on this theme.

“*If you order clinical tests for diagnosis, patients would complain that you order too many tests and want to earn money. If you tell patients that they do not suffer from a serious disease requiring specific treatment, or their disease does not belong to your professional category, then patients complain that you would not provide any treatments*.” (Chinese physician 4).

“*Chinese patients prefer to be seen by physicians they know personally. This patient also came to my department because she had a personal relationship (with my supervisor at the time).”* (Chinese physician 13).

### Inability of Patients to Understand the Risk of Complications and the Uncertainties of Medicine (Differences in Perceptions)

In both countries, physicians mentioned that patients and/or their families often did not understand the realistic risks of treatment complications, as well as the nature of medicine. Chinese physicians noted that there were disagreements that could not be resolved between the physician and patient sides. Some patients and/or their families tended to perceive any adverse event or death that occurred during medical care to be medical errors. A patient’s death was regarded as a technical problem that could and must have been addressed. Chinese physicians noted that this gap in perception could not be bridged. These physicians also stressed the uncertainties of medicine.

“*Medicine isn’t 100%. If you insist on taking 100 points to assess people, all those who got 99 points would be deemed unsuccessful*.” (Chinese physician 15).

Japanese physicians also had experiences related to this theme. They noted the difficulty of getting patients and their families to be realistic about medicine. Even after receiving detailed explanations and being well briefed on the risks of complications, when a bad outcome occurred, the patients and/or their families could not accept them and were not convinced. They had an attitude of “that’s not what I heard.” They never considered the possibility that it could happen to themselves. A physician also noted that there may be a social perception that childbirth is now completely safe in Japan.

“*In obstetrics, it is normal for a baby to be born healthy. Mothers also take it for granted that they will live. They don’t think about dying after childbirth. Thus, they cannot accept the fact that childbirth is life-threatening or disabling*.” (Japanese physician 5).

On the other hand, one Japanese physician commented that patients understand medical uncertainties to a certain degree.

“*At McDonalds, customers have a clear idea of what they can expect in terms of quality and what they get. But at hospitals, I think that patients understand, to a certain extent, that this is not the case (they cannot always get what they expect)*.” (Japanese physician 10).

### Concentration of Patients in Large Hospitals

A Chinese physician noted that large numbers of patients come to the hospital emergency room. Not only patients in urgent need of medical care but also those with only minor illnesses and non-urgent conditions visit the emergency room with their families, wreaking havoc on the medical scene. There were no direct references from Japanese physicians on this theme.

“*Actually, they didn’t have any major problems. They waste resources. We’re busy as hell, and they add to the chaos.*” (Chinese physician 17).

### Inadequate Explanation by Physicians of Treatment Plans or Potential Complications to Patients and/or Their Families

Japanese physicians noted that accountability is one of the major issues in medical disputes. Those who had faced medical disputes reflected on the sufficiency of their explanations to patients and/or their families. In one case, radiology and internal medicine departments were both involved in patient care, resulting in uncertainty about who should explain the complications of radiotherapy and, consequently, who should be held responsible if the explanations of the risks of radiotherapy were inadequate. In another case, a tense and quarrelsome family member caused the attending physician to hesitate in explaining a serious prognosis in a timely manner. Furthermore, a patient was angry with the physician who unilaterally decided the treatment strategy, including surgery, when the cancer recurred. This patient misunderstood the earliest timing of surgery due to the physician’s inadequate explanation. Physicians noted that proper and detailed explanations about pain from treatments or possible complications may have prevented a legal conflict between the physicians and the patient. There were no direct references from Chinese physicians on this theme.

“*Radiology and internal medicine were both involved in patient care, resulting in uncertainty as to who would be mainly responsible for properly explaining the complications of radiotherapy. Consequently, the patient was provided with only inadequate explanations of the risks of radiotherapy*.” (Japanese physician 1).

“*It appears that the surgeon did not fully understand how to deal with complications when they occurred*.” (Japanese physician 20).

### Misinterpretation of Physician Statements by Patients

Japanese physicians noted that misconceptions that are difficult to correct existed and led to medical disputes. One patient firmly assumed that one of the differential diagnoses was the definitive diagnosis. Some patients were confused by and sensitive to slightly different explanations they received from multiple physicians. One patient was angry because he firmly believed that surgery was delayed and this delay led to recurrence. There were no direct references from Chinese physicians on this theme.

“*I thought there are patients and families who are very sensitive to the slightest difference or nuance in statements given by more than one physician*.” (Japanese physician 18).

### Unilateral Discontinuation of the Physician–Patient Relationship by Patients

A Japanese physician stated that a patient unilaterally ended the physician–patient relationship at a university hospital and thought that this situation seemed to be a consequence of the Japanese healthcare system, which allows patients to see a doctor of their choice at a healthcare facility of their choice whenever they want. There were no direct references from Chinese physicians on this theme.

“*There is no doubt that there are some cases in which a patient says, ‘I don’t need the physician to see me anymore,’ but I don’t know if that’s a problem or not*.” (Japanese physician 3).

### Difficulty Understanding the Patient’s State of Mind by Physicians (Delayed Lawsuits)

One Japanese physician noted that, at the time of an event such as a patient’s death due to complications, the bereaved family appeared to understand and accept the physician’s explanation about the death and left the hospital. But later they sent a legal complaint to the physician. Thus, there was difficulty in determining whether the bereaved family accepted the explanation, suggesting that physicians cannot accurately judge whether family members are convinced by explanations provided by physicians about a patient’s death. Alternatively, the bereaved family may have been advised by an outsider to take the case to court. There were no direct references from Chinese physicians on this theme.

“*The physicians always say that the bereaved family understood and accepted their explanation concerning the death of a patient caused by treatment complications, but that is probably not true. I often ask the nurses about this, and they are neutral on this. I sometimes hear nurses say that the bereaved family was not convinced*.” (Japanese physician 14).

### Serious Impact of Medical Disputes on Physicians

Physicians from both countries, especially Chinese physicians, described the various ways in which medical disputes negatively impacted their mental health, professional life, and medical professionalism. These included unpleasant and negative memories that are hard to wipe away, events that will never be forgotten, negative feelings toward one’s work, strong anxiety, whether a physician continues to work (e.g., continue doing surgery), and unemployment. There were even fears of violence from patients and their families to the physician’s family members. There was also a negative impact on medical professionalism, such as defensive practices to avoid trouble with patients and/or their families, loss of will/motivation to provide the best care to patients, loss of confidence, feeling at a loss in dealing with specific patients, and strong negative feelings toward patients and/or their families, such as distrust, disrespect, and anger.

“*After the experience, you couldn’t work and didn’t want to work for a long time. Some physicians may never be surgeons again. It has a huge impact on your future career planning and career confidence*.” (Chinese physician 12).

“*I was young at the time. Even if I was scolded, I still insisted on my own ideas. But now I won’t deal with it that way, I will pretend that I don’t know, and don’t make trouble for myself. If you do that, the patient does not necessarily thank you*.” (Chinese physician 13).

“*For a long time, no matter where I went, I felt that someone was following me. Especially when I go home, I am very afraid of being followed by the patient’s family. I am worried they will hurt my family*.” (Chinese physician 19).

“*The patient’s family abused me for over an hour. It is a bad memory*.” (Japanese physician 13).

## Discussion

In the present study, we found both similarities and differences between Chinese and Japanese physicians when comparing their perceptions about medical disputes. Issues in common included monetary motives of patients and/or their families, violence/threats from patients and/or their families, inability to understand the risk of complications by patients and/or their families, and the uncertainties of medicine. Medical disputes had a serious impact on the professionalism and mental health of physicians of both countries. These findings suggest universal issues intrinsic to medicine regardless of cultural and institutional differences. Nevertheless, differences in the magnitude and frequency of these problems existed between the two countries. For instance, preexisting distrust of physicians on the patient’s side was only noted by Chinese physicians. Overall, it appeared that the work environment of Chinese physicians, including physician–patient relationships, was worse off compared to their Japanese counterparts. On the other hand, only Japanese physicians commented about insufficient information disclosure and the uncorrectable misinterpretation of physician statements by patients. This suggests that there are also major issues that differ between physicians of both countries and underlying reasons for these differences. Below, we discuss ethical implications of the cross-cultural similarities and differences between the two countries.

### Monetary Motives of Patients and/or Their Families

Physicians of both countries noted that patients and/or their families had monetary motives for medical disputes, and the number of Chinese physicians who raised this issue was greater than that of Japanese physicians. In the Japanese cases, the monetary motive was implicit and was only perceived as such by physicians, whereas in Chinese cases, the motive was explicit. One of the main reasons for this difference is presumably the complexity of the Chinese healthcare system and the heavier burden of healthcare expenditures for the general Chinese public, as shown in Table 1 and in a recent news report (Murayama 2021). Differences in insurance coverage by physician rank at the same institution or by region would also confuse patients, leading to their frustration. The high up-front costs prior to admission are a significant burden on patients and their families, making access to medical care less convenient and leaving them dissatisfied (Saito 2023).

A study which targeted the general public in China suggested that, compared to patients with social medical insurance, those with commercial medical insurance tended to evaluate physicians more positively, while those without any medical insurance tended to evaluate physicians more negatively. As medical expenses increased, physicians were rated more poorly by patients. It also became clear that there are money-related behaviors of physicians which are perceived by the public as inappropriate, and this perception affected their evaluation of physicians. Such behaviors include performing unnecessary tests and making prescriptions for money, accepting bribes from the patient side, receiving kickbacks (rebates) from pharmaceutical companies, and treating patients differently because of their social standing (Wang et al. 2022). Although the law prohibits the practice of entertaining doctors and “wrapping money in red paper,” such practices still exist (Sato et al. 2022).

Chinese people are said to have a keen sense of money (Sato et al. 2022). Many would be dissatisfied if they had to pay more or did not receive satisfactory care. If patients felt that their physician behaved inappropriately for money-related reasons and treated patients differently based on their social standing, including financial status, they may feel discriminated against and become angry, leading to medical disputes. According to one report, the relationship between medical staff and patients has been reduced to a seller-buyer interaction and patients and their family members hope to buy treatment; the authors argue that medical disputes often arise when treatment does not meet the patients’ needs or expectations as consumers (Chen and Li 2022).

Money is an important aspect in Chinese healthcare. Thus, unsurprisingly, some Chinese physicians noted that even if there was no medical fault, medical disputes would still occur. One report noted that if, despite all efforts, a patient dies, the media would suggest that all that healthcare providers are interested in is making money (Chen et al. 2020). When patients and their relatives expend their savings on healthcare but get no satisfactory results, they may find that relying on financial compensation to be the only option available. Compared with China, Japan’s medical system and national universal insurance are structured in a way that prevents medical disputes based on monetary motives.

### Violence/Threats from Patients and/or Their Families

Violent incidents or threats by patients and/or patient families were reported by physicians in both countries, but actual violence was reported only by Chinese physicians. Violence against medical professionals in China has become a significant problem in recent years (Du et al. 2020; Jia et al. 2022; Wang and Du 2023). Similarly, violence against medical professionals has become a major problem in Japan, with some physicians reportedly murdered (Ohkawara 2023; Saitama Newspaper 2022; Yasunaga 2010; Yomiuri Newspaper 2021; Yoshimura et al. 2022). Yet, in terms of the magnitude and severity of this problem, violence by patients and/or their families against Chinese physicians appears to be more serious than that against Japanese physicians.

Causes of violence to medical professionals include long waiting times and short clinic visits, dissatisfaction and distrust of the attitudes and responses of medical staff, dissatisfaction with the treatment provided and outcomes, physical and mental distress, high medical costs, requests being ignored or rejected, environmental stress, fear and uncertainty about the future, taking it out on others, lack of trust, damaged self-esteem, and being under the influence of alcohol or drugs (Chen and Li 2022; Du et al. 2020; Editorial 2019; Jia et al. 2022; Ohkawara 2023s; Yasunaga 2010). Patients’ family members also appear to be more sensitive to unpleasant stimuli at the time of visits than the patients themselves and are more likely to react violently (Yin et al. 2023). In the present study, physicians reported physical aggression based on distrust of diagnoses, violence and threats by the bereaved family at the time of the patient’s death, drunk patients taking it out on physicians, and the involvement of numerous family members.

Why does the frequency and intensity of violence and threats by patients differ between China and Japan? There are several potential reasons. First, in Japan, trust in physicians and healthcare is high, and violence and threats due to a lack of trust or dissatisfaction are less likely to occur. In a public poll (56.5% women; 8.9% aged under 29 years, 64.2% aged 30–69 years, and 26.9% aged > 70 years), 70% of respondents trusted physicians, and only 5% distrusted them. Moreover, 87% of respondents trusted the healthcare system, while only 10% did not (Murata 2022). The Chinese media outlet *Sohu* noted physicians’ high social status and respect from the public as a reason for the relative lack of medical problems in Japan compared to China (Murayama 2021). A 2018 survey of 1598 participants in China also found that 86.2% trusted doctors. However, more than half of the respondents in that survey were under the age of 20 years, more than 80% were university graduates or above, and about 70% were students, a situation which is not directly comparable to Japan. On the other hand, 54.9% of the same Chinese respondents said they would never violate or abuse a doctor, while 0.69% said they would attack or abuse a doctor (Wang et al. 2022). In another study, higher education and having medical insurance were associated with higher patient trust in their physicians (Li and Khan 2022). While it certainly is not the case that patients would never commit violence against physicians because the former trust the latter and only when trust is betrayed would violence erupt, it is likely that a strong sense of trust and respect of physicians may act as a restraint against violent behavior against physicians.

Second, Chinese people are known to value their families, and many of them respect their parents and act in the best interest of the family (Sato et al. 2022). Thus, a medical accident or unexpected death of a beloved family member would understandably incite strong negative emotions that can lead to violence. This is also reflected in the cases of the present study. While Japanese people also exhibit filial piety and collectivism due to the influence of Confucianism, they are unlikely to act as aggressively for the sake of the family for the reason discussed below (Asai et al. 2022a).

Third, compared to Japanese people, Chinese people tend to assert themselves straightforwardly and with intensity, being unafraid of confrontation (Kondo 2022; Sato et al. 2022). This tendency, combined with negative feelings and distrust that arise in the clinical setting, may manifest as violence against physicians. In contrast, Japanese people tend to avoid direct confrontation and value harmony, act with consideration for the feelings of those of higher social status than themselves, be obedient to authority, hesitate to express their feelings and thoughts directly or explicitly, worry about what others think, be concerned about inconvenience and being a burden to others, and follow laws blindly (Asai et al. 2022a, b). These psycho-cultural-social tendencies may serve as a buffer against negative emotions leading to violent behavior.

The tendency of Japanese people not to express their true feelings, thoughts, and emotions directly and hold back until the last minute could lead to the unilateral discontinuation of the physician–patient relationship by patients and the difficulty of physicians to understand the state of mind of patients. Patients tend to prefer dissolution of a relationship to direct confrontation when things go wrong, choosing avoidance rather than attack. Of course, it is also possible that physicians simply may not be capable of understanding patients or their emotional state.

### Preexisting and Strong Distrust of Physicians by Patients and/or Their Families and Concentration of Patients in Large Hospitals

These two issues, which would make work difficult for any physician, were raised only by Chinese physicians. Inevitably, it would be difficult for physicians to deal with patients and/or their families who are suspicious of all explanations regarding the need for tests and treatments, as well as diagnoses, from the first visit.

Communication is considered the most effective and efficient vehicle to engender trust. Trust—defined as “assured reliance on the character, ability, strength, or truth of someone or something”—is built over time, with repeated interactions through which expectations about a person’s trustworthy behavior can be tested (Pellegrini 2017). A recent survey in China found that physicians’ communication skills affect patient trust (Gu et al. 2022). However, a strong preexisting distrust of physicians might prevent even basic communication to begin with.

In our view, patient trust is inversely proportional to the number of patients a physician must care for within a certain period because good communication is necessary to build trust, and sufficient communication undoubtedly takes time. Since patients are not concentrated in large hospitals in Japan as they are in China, physicians at large hospitals in Japan can spend more time treating each patient (Murayama 2021). Moreover, the underdeveloped referral system in China results in the accumulation of patients in tertiary hospitals, leading to excessive workloads (Wang and Du 2023). Similarly, in outpatient clinics in Japan, where a limited number of physicians must attend to many patients within a limited amount of time, little time is available to see each patient.

Trust in physicians can be categorized as interpersonal trust and general (or public) trust. The former refers to trust in specific, identified physicians based on previous treatment relationships and the latter to trust in generalized, collective entities, i.e., physicians in general (Hall et al. 2002). The trust measured in the above-mentioned survey is general trust. Misguided and inaccurate media reports can have a strong negative impact on patient trust, resulting in a general distrust of physicians (Tang et al. 2022; Wang and Du 2023). Chinese patients have been reported to perceive hospitals in a higher tier and with a higher level of specialization as offering a better quality of healthcare; they have doubts about a general practitioner’s ability to provide medical care (Tang et al. 2022). With such preexisting views, maintaining a respectful, communicative, and trustworthy attitude toward each patient in midst of an excessive workload in order to break the distrust toward physicians would be difficult. Thus, in the current state of healthcare in China, we believe that it would be difficult to reconcile the strong general distrust of physicians through patient-physician communications, even if physicians make efforts to this end.

The general distrust of physicians in Chinese society may be related to the nature of interpersonal relationships rooted in Chinese culture. Chinese people value personal connections in their relationships. Whether or not a person is someone they know personally has a large impact on attitudes and perceptions toward that person. This is reflected in the Chinese term “*zijiren*,” which means “one of us; people on my own side, such as close friends, family members, and relatives, but not limited to blood kin” (Sato et al. 2022). This emphasis on personal connections in relationships also influences their choice of physicians. Chinese patients prefer to be treated by physicians with whom they have a personal relationship. This custom has historical and cultural underpinnings. As pointed out by a sociologist, familiarity is the prerequisite for trust (Lin 2018). Traditional Chinese society provides a good illustration of this concept. In China, where Chinese medicine has been practiced for thousands of years, medical behavior occurs within familiar social networks, and trust is gained through familiarity (Lin 2018). From our experiences, this tendency is also an ethical custom that has not changed throughout the ages, making it difficult for Chinese people to simply hand over their body and mind to a stranger. Modern medical care is new and unfamiliar, and does not have this strong foundation of trust based on patient-physician familiarity.

While it is unclear whether Chinese people are less trusting of physicians in general because of their emphasis on personal relationships, for patients, most physicians are strangers. If strangers are considered untrustworthy and this applies to physicians as well, increasing general trust of the Chinese public toward physicians may seem like an impossible task. Moreover, if Chinese people only see physicians with whom they have an established personal relationship, the opportunity to develop good relationships with other physicians will be lost, and interpersonal trust will be more difficult to establish. Unlike in many developed countries, the concept of “my physician” is not common in China, mainly due to lack of family physicians in the primary care system and the high proportion of cases which bypass the primary care system to obtain care from upper-level facilities (Li and Khan 2022; Wu and Lam 2016). Thus, the traditional culture-based relationships and the current healthcare system make it difficult to establish the concept of “my physician.” There is an analogous trend in Japan. Japanese people also distinguish between inside and outside, and have an exclusive attitude toward people outside the community to which they belong. However, the high level of trust in physicians and medical care in society likely outweighs any negative impact of this on patient consultation behavior.

### Difficulties with Explanations by Physicians and Understanding by Patients and/or Their Families

Physicians in both countries expressed the view that patients and their families were unable to comprehend medical information or understand that there were medical uncertainties and medical conditions that cannot be improved. Japanese physicians also reported on the mistaken assumptions by patients about diseases that could not be corrected. There may also be a cognitive bias at work to prevent patients and their families from accepting that death or severe complications could happen to themselves (Asai et al. 2021).

Although physicians may think that explanations of medical information they provide are sufficient, their explanations can be misunderstood, altered, or forgotten by patients. It is also possible that patients listen only to what they want to hear, or interpret information, for example, the risk of complications, in a way convenient to them. Strong preconceptions about medical care by patients get in the way of adequate understanding (Asai et al. 2021). Patients and their families may be anxious, fearful, or distressed to the extent that they lose their normal capacity to understand crucial information. For this reason, it is important for all stakeholders to reaffirm the intrinsic nature of medicine as uncertain, incomplete, and agnostic, the time constraints, and psychological situations in which patients and their families cannot fully understand medical information (Asai et al. 2022a, b). Finally, physicians should avoid judging the adequacy of information disclosure unilaterally and avoid blaming patients for their poor comprehension. Rather, they should always reflect on their own communication skills and seek appropriate disclosure methods. That said, since it is not easy for people to fully understand or empathize with each other, the problem of explaining and understanding may be a universal challenge, regardless of the country or healthcare system.

Finally, Chinese physicians did not raise issues pertaining to information disclosure, perhaps because there were many other major issues to discuss. Alternatively, they may be spending time explaining large amounts of medical information on a daily basis due to the prevalence of the buying-selling relationship in current medical practice in China (Chen et al. 2020). It is also important to note, however, that a lack of explanation and inadequate communication skills by physicians are causes of medical disputes in both China and Japan (Chen et al. 2020; Editorial 2019; Yoshimura et al. 2022).

### Serious Impact of Medical Disputes on the Well-Being, Careers, and Professionalism of Physicians

Physicians in the present study noted the various negative effects that medical disputes had on them. Much has already been reported on the negative effects of violence and conflicts on the mental state, careers, and well-being of physicians (Editorial 2019; Yin et al. 2023; Varghese et al. 2021; Wang et al. 2022). Our results also suggest that violence and threats, strong preexisting patient distrust, and the working environment of physicians had a profound negative impact on their professionalism, especially on their motivation toward providing good medical care and professional excellence in the patient’s best interest. The work environment of Chinese physicians, including physician–patient relationships, appeared to be worse off than that of Japanese physicians, and this had severe adverse effects on their morale.

According to the core medical education curriculum in Japan, physicians should be fully aware of their responsibilities as professionals who are deeply involved with human life and the protection of health, be willing to pursue their careers as physicians by respecting diversity and humanity and practicing in an altruistic manner, and be trustworthy and compassionate (Liaison and Coordination Committee for the Revision of the Model Core Curriculum 2022). We are in complete agreement with this and stress that a physician’s professionalism contributes to good physician–patient relationships, patient satisfaction, and trust in physicians.

However, adverse situations that physicians often experience can result in a distrust of people and patients, or frustration with the healthcare system, and deprive them of their original intent of entering the medical profession, leading to self-protection. Moreover, it may be difficult for physicians to trust patients and their families who do not trust physicians to begin with.

### Limitations

This study has several limitations. First, although the present study was qualitative and exploratory in nature, only limited generalizations can be made based on our results. Since only memorable cases were presented by physicians, these cases may represent the most extreme conflicts these physicians experienced and do not represent the general state of the relevant parties and healthcare circumstances of both countries.

Second, differences in background between physicians from both countries (e.g., hospital type and age, as reflected in Table 2) may also have affected the validity of our comparisons. The validity of the results could have been increased if it was possible to have recruited study participants with more similar backgrounds. Qualitative studies using sampling methods that match the backgrounds of study participants in both countries or large cross-sectional quantitative comparative studies using random sampling methods will be needed to confirm the validity and generalizability of our results. However, the findings would nonetheless be informative if physicians from both countries shared common experiences of disputes or if they suffer from similar mental health conditions, regardless of the type of hospitals to which they belong.

Third, we did not aim for theoretical saturation, and thus may have overlooked other critical issues in medical disputes. We believe, however, that we were able to conduct a comparative, qualitative cultural study with a sufficient number of participants to obtain informative results.

Fourth, since we did not ask detailed questions about the experiences, but only general ones, detailed comparisons were difficult given the lack of some factual details. As mentioned earlier, descriptions were provided unilaterally by physicians. Thus, the real perceptions and intentions of patients and/or their families were unknown, as well as how physicians, patients, and families interacted with each other. Accordingly, we did not have information on how physicians communicated with their patients, including the appropriateness of information disclosure, physicians’ attitudes toward patients and/or their family members, and whether medical errors existed or not.

Finally, interviews were conducted in Chinese or Japanese, and then analyzed and presented in English. This process could have introduced translation issues, such as misunderstanding of the details and/or true meaning of the physicians’ comments. However, given the fluency of some of the researchers in Chinese, Japanese, and English, we were able to confirm the quality and authenticity of the English translation.

## Conclusions

Based on our findings, we make the following recommendations for achieving a more harmonious physician–patient relationship, free of unnecessary conflicts, confrontations, and distrust. A generous and equitable national health insurance system and establishment of a system for sorting patient visits to prevent the concentration of patients in hospitals are essential. To the extent possible, it is also important to reduce the financial burden on patients and their families in order to prevent disputes arising from money-related reasons. Healthcare should be a public good. A fewer number of patients per physician and more time spent with each patient may increase trust in physicians and satisfaction with medical care.

While these improvements are necessary conditions, they are not sufficient. Interactions between physicians and patients and their families that develop in the clinical setting require improvement, which entails the provision of a better environment in which physicians’ professionalism can be realized. What ethical principles should physicians consider when interacting with patients in order to improve physician–patient relationships, and what should they do in order to gain interpersonal trust? Moral trust is essential to this end. Moral trust is the justified confidence of patients that physicians and their healthcare organizations are primarily committed to the protection and promotion of the health-related interests of patients and to keeping individual, group, and organizational self-interest systematically secondary, and the reliance of patients on this commitment (McCullough et al. 2020).

At the very least, we believe that the following principles are needed to establish more harmonious physician–patient relationships: (a) empathy for the suffering of patients and/or their families, given their psychological and physical vulnerability; (b) ethics, at least, of the four general principles of biomedical ethics, including respect for persons, no harm, serving the patient’s best interest, and fairness, because they are basic requirements of a good physician and, without them, patients either will not be able to survive their ailments or obtain comfortable healthcare; (c) taking emotions of patients and/or their families seriously because emotions are important for dealing with fear, anger, and distrust, and consideration of emotions is as important as clinical skills; (d) effective communication skills, given the importance of facilitating patients’ and/or their families’ understanding and appreciation of medical information, such as the need for examinations and the possibility of unavoidable treatment complications; (e) expression of positive emotions by physicians because their impassive emotions (e.g., indifference or impatience toward patients) are likely to hurt and dissatisfy patients and their families (Wang et al. 2022); and (f) education relating to all of the above-mentioned principles beginning at the undergraduate level and through postgraduate clinical training.

Notwithstanding the above principles, patients and their families can be violent and dangerous. Neither patient violence nor inappropriate media coverage can be eliminated by the efforts of physicians alone. If patients’ distrust and violence continue, even the best physician cannot properly run a clinical practice. The situation in which a benevolent physician becomes a butcher in the blinded eyes of patients and their families, leading to the latter becoming murderers at the instant of an uncontrolled emotional outburst, must be avoided. This would require raising the level of morality of people in society. Here, we use morality in the sense described by O’Neil: “morality is a set of personal and social values, rules, beliefs, laws, emotions, and ideologies collectively governing and arbitrating the rightness and wrongness of human actions” (O’Neil 2019).

We consider the most crucial point of morality here to be reciprocity. Both physicians and patients and their families need to be considerate of each other, recognizing each other’s vulnerability, rather than attempting to bend the other to one’s will by force. Violence and threats from either side are criminal acts and thus must be categorically forbidden. We need to act with decency, rather than being driven by distrust of each other, based on mutual respect, compassion, and trust from the bottom of our hearts, not just as institutional policy issues or superficial etiquette and manners. Patients and their families must be recognized as being vulnerable, and physicians must be understood to be a fragile existence with emotions and lives with their own families as well. These might be obvious concepts that go without saying, but they nonetheless can be difficult to realize. So long as these are real issues with people suffering, the importance of these concepts must be repeatedly asserted.

According to Harari, who argues that morality means reducing suffering, “we need to develop a deep appreciation of suffering. If you really understand how an action causes unnecessary suffering to yourself or to others, you will naturally abstain from it (Harari 2018).” Only truly internalized morality can bring out the warmth and sincerity in human nature, and only then can a reliable and truly harmonious physician–patient relationship be established. We believe that morality derives from our human nature, rather than commands or social codes artificially made by humans. Of prime importance now is to cultivate oneself, then achieve peace with others. In ancient China, there is a wise saying regarding management—“to cultivate oneself and to pacify others” (Shiqiang 2015).

Finally, physicians must never forget the difference in position between physicians and patients and the basic premise that patients are very vulnerable due to illness, and that physicians have voluntarily chosen to practice medicine as a profession. It is important for physicians to have a renewed and thorough understanding of their patients’ suffering and needs. However, at the same time, patients must understand the limitations of medical care (the nature of medicine), that physicians are also vulnerable, and that preconceived notions of distrust without even knowing the individual physician are inappropriate.

## Data Availability

The datasets used by and/or analyzed during the study are available for the corresponding author upon reasonable request.
